# Camera trap assessment of bushpig (*Potamochoerus larvatus*)-domestic animal interactions and implications for pathogen transmission in rural habitats of Madagascar

**DOI:** 10.1016/j.onehlt.2025.101149

**Published:** 2025-07-19

**Authors:** Rianja Rakotoarivony, Ariane Payne, Daouda Kassie, Steven M. Goodman, Alpha Andriamahefa, Modestine Raliniaina, Raphaël Rakotozandrindrainy, Ferran Jori

**Affiliations:** a UMR ASTRE (Animal-Health-Territories-Risks-Ecosystems), CIRAD-INRAE, Montpellier 34398, France; b UMR ASTRE, University of Montpellier, CIRAD, INRAE, Montpellier, France; c National Centre for Applied Research in Rural Development - Department of Zootechnical Veterinary and Fishery Research (FOFIFA-DRZVP), Antananarivo 101, Madagascar; d French Agency for Biodiversity (OFB), Research and Scientific Support Department, Orléans 45000, France; e Institut Pasteur de Madagascar, BP 1274, Antananarivo 101, Madagascar; f Field Museum of Natural History, Chicago, IL 60605-2496, USA; g Association Vahatra, BP 3972, Antananarivo 101, Madagascar; h Madagascar Institute for Vaccine Research (MIVR), University of Antananarivo, Antananarivo 101, Madagascar; i Department of Zoology and Entomology, University of Pretoria, Pretoria 0028, South Africa

**Keywords:** Ecology, Sympatry, Madagascar, Suidae, Spill-over, Pathogens

## Abstract

In some rural areas of Madagascar, bushpigs (*Potamochoerus larvatus*) are reported to be attracted to human disturbed habitats and share the same environment with domestic animals, including pigs (*Sus scrofa*). Such cohabitation can facilitate the transmission of pathogens between bushpigs and other domestic animals. To assess bushpig-domestic animal interactions and their implications for pathogen transmission, 26 camera-traps were deployed for three months around 10 villages in two separate regions of western Madagascar. The camera-traps were positioned at animal attraction sites: trophic resources, resting areas, and water points, and captured 17,804 images. No direct interactions (simultaneous presence) between bushpigs and domestic species were observed after analysis of 2678 trap nights. However, 44 indirect interactions (non-simultaneous presence) were recorded. The median critical time window (CTW), calculated as the time interval between the consecutive presence of bushpigs and some domestic species, was 646 min [34–1412 min]) for pigs, 672 min for cats [range 44–886 min], and 690 min for cattle [range 584–765 min]. Such CTW estimates are shorter than the average survival rate of several infectious pathogens potentially present in the environment, including African swine fever virus, *Mycobacterium bovis*, and *Toxoplasma gondii*. Factors such as proximity to water sources and protected areas statistically increased the chances of these interactions. Our research provided novel information on the level of interaction between bushpigs and other domestic animals in anthropized rural areas and which can be used to design and implement strategies to mitigate the risk of pathogen spread at the wildlife/livestock/human interface.

## Highlights

• First-ever study of bushpig-domestic animal spatio-temporal interactions in Madagascar, for which indirect contacts occur.
• Frequent indirect interaction between bushpigs, cattle, and cats beyond domestic pigs.
• Divergent activity patterns reduce direct bushpig-domestic pig contacts.
• Pathogen transmission theoretically possible given environmental survival times.

## 1 Introduction

The role of wild boars (*Sus scrofa*) as pathogen carriers affecting animal or human health are known since decades and well described in the literature [1,2]. Their presence in human-modified environments and interactions with other domestic animals, particularly domestic pigs (DP) can result in pathogen spill-over to humans or domestic animals [2]. In contrast, studies addressing interactions between wild pigs and domestic animals in sub-Saharan countries are limited [3,4]. The bushpig (BP, *Potamochoerus larvatus*), together with the closely related red river hog (*P. porcus*), native to eastern/southern Africa, represent some of the most widespread Suidae species in sub-Saharan Africa [5]. They are attracted to human-modified habitats and both are widely hunted for their meat and to protect crops from their crop-raiding behaviour [3,4]. On Madagascar, the BP, introduced an estimated 2000 years ago, inhabits forests, open grasslands with scattered trees, and crop fields [6], becoming one of the island's largest land mammals after Quaternary megafauna extinctions [7]. Today, free-ranging DP are common and widespread on the island [8], increasing habitat overlap and interaction risks with BP.

Camera traps (CT) are widely used to quantify interspecific interactions, for example between domestic and wild animals, and provide the means to assess sympatric utilization of the same areas and contact rates. By accounting for pathogen survival time in the environment under varying weather conditions, critical time windows (CTW) can be identified. Once defined, CT data can be used to estimate contact rates that may facilitate pathogen transmission within these CTW [9]. Nevertheless, this approach has been seldom applied in the context of *Potamochoerus* species, for which data on their capacity to carry infectious pathogens are limited. BP are known to be resistant to African swine fever virus (ASFV) infection [10] and suspected to act as reservoirs for this virus. Although experimental infections show BP can carry ASFV asymptomatically and excrete it for 35–91 days [10], their role in ASF epidemiology remains unclear [[10], [11], [12]]. In addition, on continental Africa, BP have occasionally been found infected or exposed to some zoonotic pathogens such as *Mycobacterium bovis* and *Toxoplasma gondii* [13,14]. On Madagascar, a recent study based on participatory methods with resident stakeholders reported the occurrence of direct and indirect interactions between BP and DP in two western regions of the island [15]; these inferences were based on local knowledge. Therefore, the goal of this study was to characterize and quantify the frequency, spatial distribution, and temporal patterns of interactions between BP and other domestic animal species in these same areas. The study focused on the quantification of direct or indirect interactions to infer a potential risk of pathogen transmission between sympatric BP and domestic species. Based on available litterature, we considered potentially circulating between BP and DP include ASFV, but also others circulating at the wildlife/livestock human interface such as *M. bovis* or *T*. *gondii*.

## 2 Material and methods

### 2.1 Study site

**Fig. 1:**
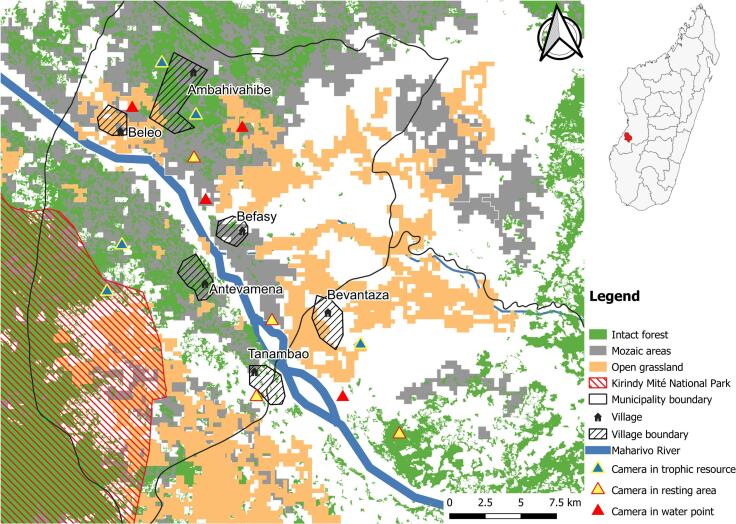
Map of field sites for camera trap survey in the Menabe Region.

**Fig. 2:**
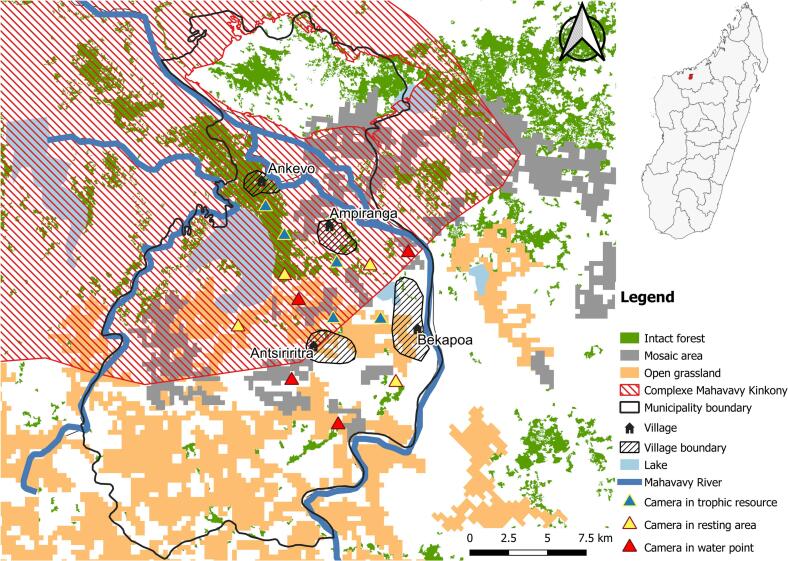
Map of field sites for camera trap survey in the Boeny Region.

Our study was conducted in two areas of western Madagascar, specifically the Menabe Region and Boeny Region, separated by 450 km (Fig. 1 and Fig. 2). Both regions include a mosaic of different vegetation types, as well as the presence of protected areas including the Paysage Harmonieux Protégé du Complexe Zones Humides Mahavavy Kinkony [16] in the Boeny Region and the Parc National de Kirindy Mité [17] in the Menabe Region. The selected sites in Boeny are located on average 5 km from the eastern edge of the Mahavavy Kinkony protected area and encompassed mangroves, dry deciduous forests, and palm grassland interspersed with secondary thickets. Selected sites in Menabe are located on average 15 km to the northeast of the Kirindy Mité National Park and included large intact and degraded dry deciduous forests, and grasslands.

The main sources of income for villagers in these regions are agricultural production of rice, maize, and cassava, and to a lesser extent sugar cane and groundnuts. Most households raise domestic animals such as poultry (chickens and ducks), pigs, and cattle (mostly in free-ranging conditions), as well as dogs and cats.

### 2.2 Study design

In each study area, we identified multiple zones based on free-range DP farming, forest cover abundance, proximity to protected areas, and cultivated crops. A previous study using participatory mapping and focus group discussions, highlighted areas of potential interaction between DP and BP [15], guiding our selection of 13 CT deployment sites per study area. Site selection prioritized accessibility, CT security, and landowner acceptance, with sites further classified into three types—trophic resources, water points, and resting areas—based on the presence of suspected BP and DP attractants. Trophic resources included fruit trees (mango, *Mangifera indica*; mokotro, *Strychnos spinosa*; jujube, *Ziziphus jujube*; raffia, *Raphia farinifera*) and crops (rice, corn, cassava, sugar cane, and groundnuts). Resting areas consisted of bushes and mires identified by local stakeholders as common resting sites for both suids and included locations different from water sources and trophic resources.

### 2.3 Camera trap surveys

We conducted CT surveys for 3 months (encompassing a total 90 days) of the dry season (August–October) at each study area—Menabe in 2022 and Boeny in 2023. This period coincided with the ripening of certain wild fruits (jujube, mokotra, raffia), harvesting of crops (rice, maize, groundnuts, and cassava), and a high level of BP foraging activity [18]; it also served as a suitable period for free-ranging DP [19].

The 26 infrared motion-triggered cameras (Bolyguard SG-2060X) were deployed 40 cm above ground level, set to capture three photos per trigger with a 10 s delay. Cameras operated continuously, monitored weekly for maintenance, and no baits or lures were used.

Each site was separated from the nearest one an average of 2.5 km (SD = 1.6 km) apart; this distance was chosen based on reports of the average distance travelled by BP and DP during the course of a day [20,21] and consisted of two CT, in case of malfunctioning of one of the two cameras. We recorded GPS location of each deployment site using a handheld GPS device (Garmin Etrex 30; Garmin International Inc., Olathe, KS, USA) and georeferenced in Universal Transverse Mercator (UTM) coordinates using World Geodetic System Datum 84 (WGS84) projection.

### 2.4 Image classifications and variable definitions

Overlapping photos were analyzed to remove duplicates and false detections. Species were identified using a machine learning process for animal species recognition and classification [22], combined with manual verification. An independent capture event was defined as a photograph of the same species taken by the same CT more than 30 min apart [23]. Trap-nights (24-h periods) measured trapping success (captures/trap-nights×100) to assess species activity levels.

A direct interaction was defined as the simultaneous presence of BP and another species in the same photograph. An indirect interaction was defined as two consecutive visits of BP and another species within a specific Critical Time Window (CTW). We used a conservative CTW of 24 h based on the estimated shortest persistence time of still viable ASFV in tropical environments [24]. Time intervals in minutes between visits of two species were used to calculate bidirectional contact rates facilitating pathogen transmission expressed by mean, median, and maximum-minimum ranges.

Deployment sites were classified into three habitat types: intact forest (dense canopy, rich understory), open grassland with scattered trees, and mosaic areas (transitional between forest and open grasslands). This categorization helped to assess habitat influence on species presence and visitation patterns, reflecting varying ecological preferences and adaptations.

Due to the low number of observed indirect interactions, the analysis of interaction drivers was conducted using the combined data from both study areas. Additional explanatory variables were incorporated in our analysis, including human and DP population estimations per village [25]. Vegetation and land use data were collected via participatory mapping, site visits, and shapefiles from Data Basin site [26], and OpenStreetMap [27]. Distances to water sources and protected areas were measured using handheld GPS and Quantum Geographic Information System (QGIS), integrating field and geospatial data for analysis (Supplementary file 1, Table S1).

### 2.5 Statistical analysis

We used non-parametric kernel density estimation to analyze DP and BP activity patterns using the R package “overlap” [28]. Temporal overlap coefficients (Δ_1_ for small datasets, Δ_4_ for large datasets) were calculated for four different temporal periods (dawn, dusk, day, and night) ranging from 0 (no overlap) to 1 (complete overlap). Accuracy was assessed via bootstrapping and the “activity” package [29]. Activity patterns were compared using Watson-Wheeler's *U*^*2*^ test (“circular” package) for significant differences [30].

To avoid collinearity, each variable was tested individually in linear regression models, omitting those with a variance inflation factor (VIF) > 2 [31]. Over-dispersed (number of interactions) count data were analyzed using negative binomial models, with CT nights as an offset and camera ID as a random effect (glmer.nb, “lme4” package) [32]. Models were ranked using AICc (“MuMIn” package) [33], and incidence rate ratios were reported for significant variables (ΔAICc <2). Log-transformed time intervals between BP and DP visits were analyzed using linear regression (“MASS” package) [34]. For model screening, we selected a bidirectional stepwise approach, incorporating both forward variable selection and backward elimination at each step. All analyses were conducted in R [35], with α = 0.05 for significance.

## 3 Results

### 3.1 Species detection and habitat use

A total effort of 2678 trap-nights (median 36 nights/site, range 12–42 nights/site) in 26 CT deployment sites resulted in 1755 photographs of various animal species. Species were classified into nine groups based on taxonomic relatedness and ecological similarity (e.g., domestic vs. wild, suids vs. birds). Domestic (largely chickens, *Gallus gallus*) and wild fowl (mostly helmeted guineafowl, *Numida meleagris*) were the most detected species, followed by BP, DP, and free-ranging cats (*Felis catus*) (Supplementary file 1, Table S2).

**Fig. 3:**
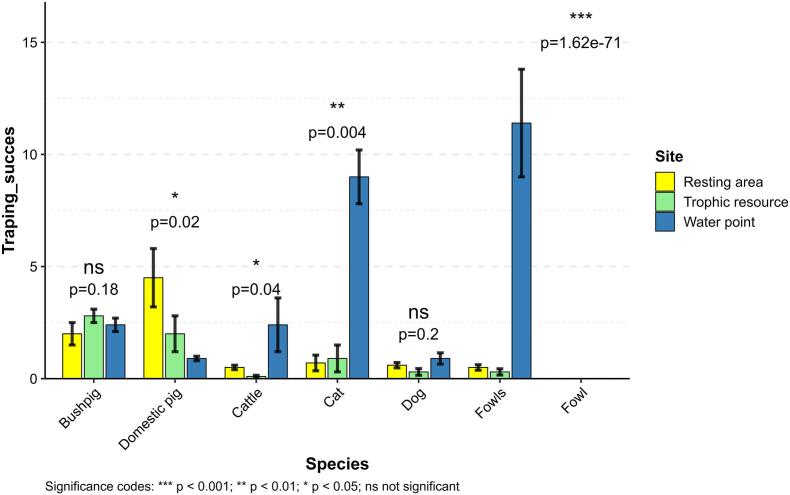
Averages of the trapping success (and standard errors) for the main species detected across the different types of deployment sites in the Boeny and Menabe Regions.

The visitation frequency of BP was not significantly different (*p =* 0.18), across the three types of deployment sites (resting area, trophic resources, and water points). In contrast, DP displayed a significantly higher frequency of visits to sites within trophic resources, when compared to the resting area and water point sites (*p =* 0.02), with trapping successes of 4.5, 2.0, and 0.9 capture/trap-nights x 100, respectively. Cameras also captured other domestic species such as cattle (*Bos indicus*) and dogs (*Canis familiaris*). Cattle were much more common at water points (*p =* 0.04) (Fig. 3), while no difference was observed for dogs (*p =* 0.2). Cats were more frequently detected at water points compared to the other sites, resulting in a trapping success eight times higher than the other two site types (i.e. 9.0 vs. 0.9 in trophic resources vs 0.7 trapping success in resting area, respectively) (*p =* 0.004). Fowl showed significantly different visitation frequencies across the three types (*p* < 0.0001), with detection rates being markedly higher at water points compared to both resting areas and trophic resource sites. There was no significant difference in the number of capture events for each species between the two regions (all *p-*values >0.1).

### 3.2 Temporal activity patterns

**Fig. 4:**
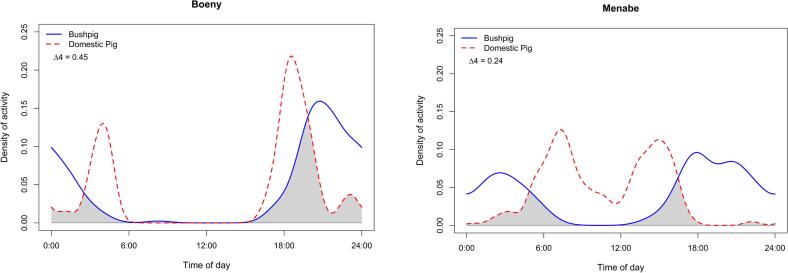
Density estimates of temporal activity displayed by BP and DP in the Boeny and Menabe Regions. Activity overlap between species is represented by the gray shaded area between lines. Delta Δ_4_ is the most appropriate overlap coefficient.

In both study site regions, BP were primarily nocturnal, active between 13:00 and 06:00, with peak activity observed at 18:00. In the Menabe Region, DP were diurnal and active between 06:00 and 18:00, with peak activity at 08:00. In the Boeny Region, DP activity peaked around 18:00, displaying a diurnal activity mostly concentrated after 14:00. The majority of indirect interactions between these two suids took place between 6:00 and 18:00 (Supplementary file 1, Fig. S1). We found moderate temporal overlap in activity between BP and DP in the Boeny Region (Δ_4_ = 0.45, 95 % CI 0.32–0.58) and in the Menabe Region (Δ_4_ = 0.24, 95 % CI 0.17–0.34) (Fig. 4). Both species showed significant differences in temporal patterns within each region (Boeny Region: U^2^ = 1.29, *p* < 0.01; Menabe Region: U^2^ = 3.06, *p* < 0.01).

### 3.3 Interactions between Suidae species

We recorded no direct interaction between the two Suidae species based on the CT data. However, considering a CTW of 24 h, we recorded a total of 18 indirect interactions in Boeny and 26 in Menabe (total = 44). The number of indirect interactions at each camera deployment site varied from 0.0 to 0.1 events per trap-night (mean = 0.01). Considering the number of capture events and trapping success rates, there was a significant difference in the number of indirect interactions recorded between the two regions (*p* = 0.03).

**Fig. 5:**
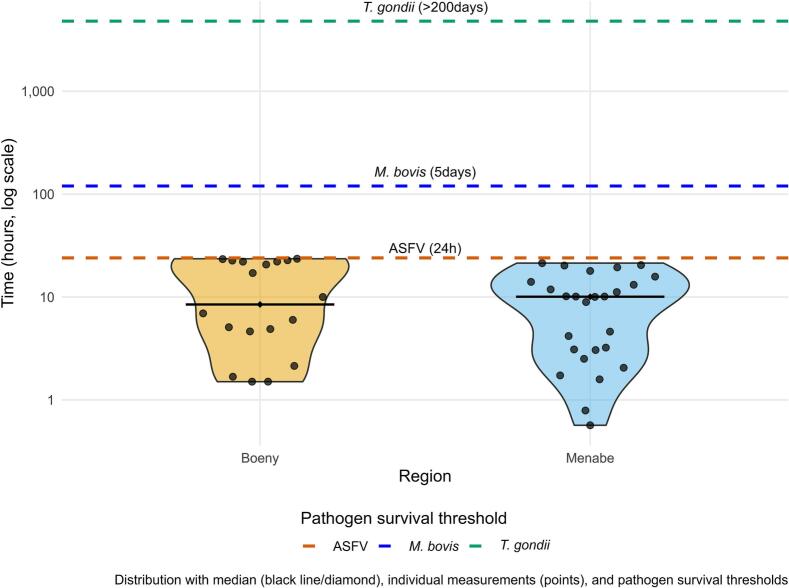
Temporal overlap analysis of visit intervals and pathogen persistence. Violin plots show the distribution of intervals (hours) between visits by BP and DP at shared sites, compared to environmental survival times of [24], *Toxoplasma gondii* [46], *Mycobacterium bovis* [49], and the observed CTW.

After excluding models based on variable collinearity, the best-supported model showed that indirect interactions between Suidae species increased near villages and protected area boundaries, influenced by site type and vegetation cover (*w*_i_ = 0.47) (Supplementary file 1, Table S3). Interaction rates were 3.47 times higher within 1 km of villages versus >2 km. Water points significantly boosted interactions (*p* = 0.001), while trophic resource sites saw a 43 % increase compared to resting points. Open grassland-bordered sites had 1.42 times higher interaction rates than other vegetation types (Table 1). Median inter-visit intervals for suids were 646.54 min (Supplementary file 1, Table S4), showing no regional variation (*p* = 0.6). Fig. 5 compares the distribution of visit intervals between BP and DP with the environmental persistence periods of three pathogens: ASFV (24 h [24]), *T. gondii* (>200 days) [46], *M. bovis* (5 days [49]). Regarding the visit intervals, the final model showed that proximity of water source was associated with shorter visit intervals between Suidae species (*p =* 0.02) (Table 2).

**Table 1:** Estimates and Incidence Rate Ratios (IRR) of the variable associated with the number of indirect interactions in Boeny and Menabe regions.

Predictors	Estimate (SE)	IRR [95 % CI]	Z value	Pr (>|z|)
Distance nearest village to protected area a boundary				
More than 2 km from the boundary of the protected area		Reference		
Between 1 and 2 km from the boundary of the protected area	0.08 (0.79)	0 [0.00–4.07]	0.98	ns
Less than 1 km from the boundary of the protected area	1.78 (1.82)	3.47 [3.18–44.47]	0.11	*

Site characteristics				
Resting area		Reference		
Trophic resources	4.79 (3.01)	1.43 [0.88–5.17]	0.98	**
Water points	2.13 (2.07)	2.08 [1.25–7.45]	1.42	***

Vegetation type cover in each deployment site				
Forest		Reference		
Open grassland	0.09 (1.73)	1.42 [0.01–14.00]	0.05	**
Mosaic area	2.18 (0.60)	8.09 [2.92–48.52]	0.97	ns

*p*-values: ns, *p* > 0.05/ *p < 0.05/ **p < 0.01/ ***p < 0.001.

**Table 2:** Linear regression evaluating the predictor variables related to the interval between visits of BP and DP at a deployment site and with combined data from both study regions.

	Estimate (SE)	*t* value	Pr(>|t|)
(Intercept)	0.71 (1.10)	0.65	0.52
Distance between site and water source	1.45 (0.33)	4.29	0.02⁎

⁎ *p*-value < 0.05.

## 4 Discussion

Repeated mention has been found in the literature of *Potamochoerus* spp. interacting with sympatric free-ranging DP populations at different locations on the African continent [36,37]; however, these events have rarely been analyzed and quantified. A previous study in Uganda using CT attempted to address this question, but lacked a sufficient number of observations to quantify interactions [4]. To our knowledge, the data presented herein are the first providing quantitative information on the frequency of ecological interactions between *P. larvatus* and sympatric domestic animals. We observed significantly greater activity overlap between the two suid species in Boeny than in Menabe (*p* = 0.03). This difference may be attributed to the larger protected area adjacent to our Boeny study site [16], as well as a higher concentration of CT within the protected area in this region. Furthermore, the levels of deforestation and settlement in Boeny, specifically inside the Mahavavy Kinkony protected area were higher [16], as compared to Kirindy Mité in Menabe [38]. Therefore, habitat destruction might influence populations of BP to venture further into agricultural areas in search of food, increasing the likelihood of encounters with DP. In each region, the fact that both pig species shared the same ecological habitats and resources but were active during different temporal periods (mainly diurnal versus nocturnal), combined with the lack of direct interactions, indicates that immediate encounters between both species are probably rare.

Despite our study design tried to maximize the chances to observe direct interactions between the two Suidae species, not a single case was detected. This can be explained by the divergent activity patterns of each of these species. This result is only partially consistent with previous information collected through interviews with local stakeholders (farmers and hunters) in the same areas of Madagascar, who reported the occurrence of sexually driven direct interactions. More specifically, based on these interviews, it was suggested that the interactions were between BP males and sexually receptive domestic sows during the months of June to October, which coincide with the BP breeding season. Considering the shortest time period, based on the CT data, measured between both Suidae species (34 mins) and the limited spatio-temporal window of observation of our study, the possible occurrence of sexually driven interactions cannot be completely ruled out. Further investigations with more powerful ecological tools (telemetry, proximity loggers, or more intensive CT deployments) could potentially shed some light on the occurrence of occasional direct BP-DP interactions.

Our regression model identified trophic resources and water points as key attraction hotspots for both pig species, and associated with indirect interactions (Table 1); parallel results have been found in Uganda [4]. Fruit-bearing trees (mango, jujube, etc.) were an important attractant in our study. Indirect interactions peaked near water sources [3] and crops, likely due to declining forest resource availability during lean seasons forcing these animals to seek alternative food and water sources in human-modified landscapes [39,40]. Open grassland habitats also significantly favored indirect interspecific interactions, corroborating a study in a nearby are linking BP aggregation to *Strychnos spinosa* and *Ziziphus jujuba* fruiting [18]. Another of our relevant findings was that indirect interactions were more significantly common as the distance between the protected areas boundary and the nearest villages decreased. This is consistent with BP being more abundant at the ecotone between protected area boundaries and nearby agricultural areas [41] and with parallel results found in Uganda [4].

Frequent indirect interactions between sympatric species can lead to interspecific transmission of shared pathogens. We verified the likelihood of potential interspecific transmission between species by comparing median times of the CTW of BP and DP, superimposed on the environmental survival times for potential circulating pathogens found in available published literature.

Despite studies documenting the circulation of infectious pathogens in *Potamochoerus* species are limited, one of the most important ones is ASF for which BP are considered a potential natural reservoir [10]. In Eurasia, wild boars infected with ASFV have the capacity to excrete the virus in the environment [42,43]. Assuming that ASF infected BP have the same capacity and given the environmental stability of ASFV in contaminated soils [44,45], infected BP fecal matter could potentially maintain viral persistence in the environment. Based on the observed CTW between BP and DP (24 h), our data suggests sufficient time for transmission of ASF through their sympatric occurrence in the same contaminated environment.

In addition, a CTW of 24 h would also be sufficient to allow potential transmission of other environmental pathogens between species using the same habitat. Indeed, a total of 32 indirect interactions between BP and domestic cats were also recorded (mean visitation interval = 11.2 ± 4.3 h). Considering that cats are the natural reservoir of *Toxoplasma gondii* and that oocysts from infested mammals can persist in the environment for more than 200 days [46], these interactions could contribute to interspecific transmission at the interface between natural forest and human-disturbed habitats, as has been shown for endemic and threatened forest-dwelling Malagasy carnivores [47]. Moreover, free-ranging and wild pigs are particularly exposed to oocysts of *T. gondii* potentially present in the contaminated environment when rooting the soil. They can also get exposed to tissular bradyzoites when scavenging on carcasses [48]. In the specific case of BP, seroprevalence study recently conducted in Parc National de Makira, a rainforest in northeastern Madagascar, detected for the first-time antibodies against *T. gondii* in this species [Raharinaivo et al., 2024 submitted]. Even in the absence of currently available data on circulation of *T. gondii*, published available information strongly suggests that our study area is favorable to the circulation of this pathogen. Nevertheless, further studies are encouraged in order to confirm this hypothesis.

On the basis of our observations, another multi-host pathogen potentially circulating in our study area is *Mycobacterium bovis.* This bacterium is the causative agent of bovine tuberculosis (bTB) in cattle and wildlife [13,14]. In our study a total of 29 indirect interactions between BP and cattle were recorded, with an average visit duration of 11.5 h (± 6.8). In the tropical dry environments of our study sites, *M. bovis* could survive for five days [49]. This pathogen has been reported to actively circulate in cattle in rural zones of Madagascar [50] and is known to infect BP and other wild suids in eastern and southern Africa [13,14]. Again, these references combined with our findings provide a strong basis to suspect that our study area is suitable for potential spill-over and shared circulation of *M. bovis* between wild and domestic species but further studies should be conducted to provide local evidence of transmission or exposure between different hosts in our identified interaction hot-spots. Of particular concern in the context of sympatry between wild and domestic hosts in anthropized habitats is the widespread occurrence of human practices. Our results highlight how anthropogenic landscape features—particularly proximity to villages and water points (number of interactions 2.08 times higher than in resting area)—create hotspots for potential zoonotic transmission. These high-risk zones amplify concerns about human practices that facilitate exposure routes which include contaminated water, consumption of raw milk or improperly cooked meat, and the lack of hygiene when or after manipulating carcasses of wild or domestic animals [13]. Similarly, the lack of proper disposal of carcasses of wild or domestic animals or their remains after slaughter can facilitate cross contamination between free-ranging animal hosts.

To mitigate these risks, integrated surveillance systems should be established to enable early pathogen detection. From that perspective, our study should be useful to identify areas for this targeted surveillance near identified interaction hot-spots or in locations with similar characteristics. In addition, other targeted interventions might include awareness campaigns among local communities to promote safer practices for meat handling, carcass disposal, milk consumption and hygiene practices, as well as recommendations to prevent zoonotic exposure and reduce environmental contamination.

## 5 Conclusions

This is the first study investigating potential interactions between BP and domestic animals in two different areas of rural western Madagascar. The results at both sites were highly consistent and found mainly indirect interactions between BP and DP, as well as cats and cattle. Divergent activity patterns between BP and DP suggest that direct contacts between both Suidae species are unlikely to occur unless under exceptional circumstances. Based on available data on environmental survival times of some zoonotic pathogens, the observed time intervals between the presence of BP and the identified domestic species, was compatible with the potential transmission of pathogens affecting animal health, but also environmental and human health at the wildlife/livestock/human interface. This situation calls for an urgent One Health approach to monitor well-being at the human-animal-environment interface, promote cross-sectorial collaborations (veterinary, public health, ecology), implement risk reduction strategies such as awareness campaigns among local rural communities, and integrated surveillance activities.

## CRediT authorship contribution statement

**Rianja Rakotoarivony:** Writing – review & editing, Writing – original draft, Visualization, Validation, Supervision, Software, Resources, Project administration, Methodology, Investigation, Formal analysis, Data curation, Conceptualization. **Ariane Payne:** Writing – review & editing, Writing – original draft, Visualization, Validation, Supervision, Methodology, Formal analysis, Conceptualization. **Daouda Kassie:** Writing – review & editing, Writing – original draft, Visualization, Validation, Supervision, Software, Resources, Project administration, Methodology, Formal analysis, Data curation, Conceptualization. **Steven M. Goodman:** Writing – review & editing, Writing – original draft, Visualization, Validation, Software, Methodology. **Alpha Andriamahefa:** Visualization, Supervision, Resources, Investigation, Data curation. **Modestine Raliniaina:** Writing – review & editing, Writing – original draft, Visualization, Resources, Project administration, Investigation, Conceptualization. **Raphaël Rakotozandrindrainy:** Validation, Supervision, Project administration, Conceptualization. **Ferran Jori:** Writing – review & editing, Writing – original draft, Visualization, Validation, Supervision, Resources, Project administration, Methodology, Funding acquisition, Formal analysis, Data curation, Conceptualization.

## Funding

This study was supported and financed by the Ecology and Evolution of Infectious Diseases Program, grant no. 2019–67015-28981 from the USDA—National Institute of Food and Agriculture, in a project entitled “Unraveling the effect of contact networks and socioeconomic factors in the emergence of infectious diseases at the wild-domestic interface” (https://www.asf-nifnaf.org/).

## Declaration of competing interest

The authors declare that they have no known competing financial interests or personal relationships that could have appeared to influence the work reported in this paper.

## Data Availability

The data that support the findings of this study are available on reasonable request from the corresponding author, R. Rakotoarivony.
