# Supplementary material for: Camera trap assessment of bushpig (Potamochoerus larvatus)-domestic animal interactions and implications for pathogen transmission in rural habitats of Madagascar
Source: One Health. 2025 Jul 19;21:101149. doi: 10.1016/j.onehlt.2025.101149 (PMC12309490; doi:10.1016/j.onehlt.2025.101149)
Supplement: Supplementary material [file mmc1.docx]

**Supplementary materials**

Table S1: List of variables used in the camera-trap analyses.

| Variables | Categories | Source |
| --- | --- | --- |
| Characteristics of each deployment site | Resting area Trophic resource Water points | Site visit Participatory mapping  Participatory mapping |
| Distance from deployment site to water source | Numeric variable | Geographic coordinates Open Street Map OSM <https://openstreetmap.org/> |
| Distance village to protected area | [0 – 1 km [ [1– 2 km [ More 2 km | Geographic coordinates Madagascar Biodiversity Network REBIOMA  <https://www.rebioma.org/index.php/fr/2014-05-30-08-40-13/telechargement/cat_view/14-gis-data> |
| Vegetation type cover in each deployment site | Intact forest Open grassland Mosaic area | Madagascar Biodiversity Network REBIOMA  <https://www.rebioma.org/index.php/fr/2014-05-30-08-40-13/telechargement/cat_view/14-gis-data>  Conservation Biology Institute  <https://databasin.org/datasets/dda466e5b9df443390a7714805e3137e/> |
| Human population in each village near the deployment site | [0 – 1 km [ [1 – 2 km [  More 2 km | Institut National de la Statistique (INSTAT), 2020 <https://www.instat.mg/documents/upload/main/INSTAT_RGPH3-Definitif-ResultatsGlogaux-Tome2_17-2021.pdf> |
| Pig population in each village near the deployment site | [0 – 50 [  [50 –100 [  More 100 | Census of the pig population by veterinarian official and para-veterinary agents in both study regions. |

Table S2: Number of capture events and trapping success rates (number of capture events per trap-night x 100) of species detected by camera traps in both regions. * before scientific name indicates introduced to Madagascar

| Vernacular name | Scientific name | Capture events | Trapping success |
| --- | --- | --- | --- |
| Bushpig | **Potamochoerus larvatus* | 217 | 8.10 |
| Domestic pig | **Sus scrofa* | 197 | 7.36 |
| Cat | **Felis catus* | 122 | 4.56 |
| Dog | **Canis familiaris* | 49 | 1.83 |
| Cattle | **Bos indicus* | 82 | 3.06 |
| Small ruminants (goat & sheep) | **Ovis aries*  **Capra aegagrus hircus* | 11 | 0.41 |
| Domestic and wild fowl | **Gallus gallus*, *Numida meleagris* | 310 | 11.58 |
| Small mammals  (rodents, insectivores, small lemur) | **Mus musculus*, *Tenrec ecaudatus*, *Microcebus* sp. | 24 | 0.90 |
| Wild carnivores | *Cryptoprocta ferox*, **Viverricula indica* | 22 | 0.82 |

Table S3: Candidate model for variables expected to predict the number of indirect interactions between BP and DP

| Candidate model | AICc | ΔAICc | *w_i_* |
| --- | --- | --- | --- |
| Distance of the village from the boundary of the protected area + Characteristics of each deployment site + Vegetation type cover | 254.93 | 0.00 | 0.47 |
| Distance of the village from the boundary of the protected area + Distance deployment site to water source + Characteristics of each deployment site + Vegetation type cover | 255.87 | 0.94 | 0.30 |
| Distance deployment site to water source + Characteristics of each deployment site + Vegetation type cover | 257.91 | 2.97 | 0.11 |
| Distance of the village from the boundary of the protected area + Human population + Characteristics of each deployment site + Vegetation type cover | 258.32 | 3.38 | 0.09 |
| Distance of the village from the boundary of the protected area + Distance deployment site to water source | 262.19 | 7.26 | 0.01 |
| Vegetation cover | 262.98 | 8.05 | 0.01 |
| Distance of the village from the boundary of the protected area + Vegetation type cover | 263.35 | 8.42 | 0.01 |
| Human population + Vegetation type cover | 264.18 | 9.25 | 0.00 |
| Distance deployment site to water source + Characteristics of each deployment site + Vegetation type cover | 272.26 | 17.33 | 0.00 |
| Distance deployment site to water source + Vegetation type cover | 272.90 | 17.97 | 0.00 |

ΔAICc Difference between each model Akaike information criterion (AIC) value and the one of the lowest AIC

*w_i_* Akaike weight of the model

Table S4: Number of indirect interactions, median time (in minutes), and interquartile intervals of indirect interactions between BP and DP in the two regions of this study.

| Region | Nb. Interactions (BP-DP) | Median | IQR |
| --- | --- | --- | --- |
| Boeny | 18 | 508.25 | 281.69 – 1325.64 |
| Menabe | 26 | 604.32 | 183.86 – 829.25 |

Figure S1: Distribution of the indirect interaction between BP and DP during diel cycle. Blue steps are observed frequencies, continuous green line being the mean.
